# Pleasure and Addiction

**DOI:** 10.3389/fpsyt.2013.00117

**Published:** 2013-09-25

**Authors:** Jeanette Kennett, Steve Matthews, Anke Snoek

**Affiliations:** ^1^Department of Philosophy, Macquarie University, Sydney, NSW, Australia; ^2^Australian Catholic University, Sydney, NSW, Australia

**Keywords:** addiction, pleasure, autonomy, choice theory, reward and motivation

## Abstract

What is the role and value of pleasure in addiction? Foddy and Savulescu (1) have claimed that substance use is just pleasure-oriented behavior. They describe addiction as “strong appetites toward pleasure” and argue that addicts suffer in significant part because of strong social and moral disapproval of lives dominated by pleasure seeking. But such lives, they claim, can be autonomous and rational. The view they offer is largely in line with the choice model and opposed to a disease model of addiction. Foddy and Savulescu are sceptical of self-reports that emphasize the ill effects of addiction such as loss of family and possessions, or that claim an absence of pleasure after tolerance sets in. Such reports they think are shaped by social stigma which makes available a limited set of socially approved addiction narratives. We will not question the claim that a life devoted to pleasure can be autonomously chosen. Nor do we question the claim that the social stigma attached to the use of certain drugs increases the harm suffered by the user. However our interviews with addicts (as philosophers rather than health professionals or peers) reveal a genuinely ambivalent and complex relationship between addiction, value, and pleasure. Our subjects did not shy away from discussing pleasure and its role in use. But though they usually valued the pleasurable properties of substances, and this played that did not mean that they valued an addictive life. Our interviews distinguished changing attitudes towards drug related pleasures across the course of substance use, including diminishing pleasure from use over time and increasing resentment at the effects of substance use on other valued activities. In this paper we consider the implications of what drug users say about pleasure and value over the course of addiction for models of addiction.

Well don’t get me wrong, I love using mate. If I could use successfully I would. I’d still be using. I love using. I just don’t like the shit that comes with it. (R50)

## Introduction

According to the so-called Moral Model (or Lay View), held by many, perhaps the majority of ordinary people, drug use by people who satisfy standard definitions of addiction is not the product of a disease or disorder that undermines the autonomy of the user. Drug use is voluntary behavior motivated by pleasure. The Lay or Moral Model of addiction takes a stern normative stance on the seeking of pleasure in this way – it regards it as parasitic, irresponsible, hedonism.

The minimal Liberal view of addiction expounded by Foddy and Savulescu (1) rejects the moralism of the Lay view but agrees with it that drug use in addicts is voluntary, pleasure-seeking behavior, and that we can draw no adverse conclusions about the autonomy of addicts from their repetitive drug-seeking behavior. In arguing against neurobiological versions of the Disease Model of addiction they say this:

In plain English, if we repeatedly obtain some pleasurable experience we start to want it more. It moves up the rankings of experiences we would like to repeat. If we regularly engage in an extremely pleasurable experience, it is only natural that we will come to place a higher importance on that experience. The Liberal View is not so minimal that it cannot say what addictions are. They are strong appetites toward pleasure. (2010: p. 15)^1^

On their view, although an addicted person may well periodically regret his addictive behavior, he nevertheless at the moment of consumption acts in order to satisfy an appetite for pleasure and this choice is not obviously either irrational or lacking autonomy.

The Lay and Liberal positions provoke an inquiry into the relation between pleasure and addiction; in particular, they provoke us to consider what role pleasure plays in the moral psychology of the addicted agent. Are addicted persons motivated by pleasure alone? And is pleasure the object of their actions throughout the course of their addiction?

We will address these questions in two ways: first, we will examine the Choice account of motivation that we take to underlie the Liberal View to test the status of the claim that addictive motivation can be explained in terms of pleasure-seeking. What is meant by this claim and what would count as evidence against it? Second, we will probe the role that pleasure plays in addictive actions via evidence sourced from addicted persons themselves. In a recent study (*n* = 69), semi-structured interviews were undertaken to explore the effects of substance use on what addicted persons value, specifically in relation to the ways addiction has impacted on the course of their lives. During these interviews many of our subjects offered accounts of the phenomenology of addiction and addictive motivation. Our research indicates that there are important nuances to the role that pleasure plays in addiction; that changes in motivations for substance use occur over the course of addiction; and that there is variation in how pleasure itself is evaluated by the individuals concerned. We hope in these two ways to supply a richer and more informative account of motivation in addiction than is currently accepted by holders of either the Lay or Liberal view. Our results, together with other evidence, suggest that addictive motivation is complex, and make it doubtful that severely addicted persons’ consumption can be subsumed under the category of ordinary weakness of will.

## Choice Models of Addiction

Recently, models of addiction which arise from behavioral economics and the psychology of choice have taken center stage in the ongoing debate over how best to characterize what goes wrong in addiction. Choice theorists see this model as breaking the impasse between Medical models (including the brain disease model), which remove or diminish, perhaps unacceptably, the agency of those who are addicted, and Moral or Lay models which condemn them. Disease models claim that the behavior of addicts is substantially involuntary – that it is caused by processes which bypass deliberation and choice or that are impervious to them. Moral models deny this and claim that the goals and values of the addicted person are bad or their choices and actions are weak in ways which reflect poorly on them.

By contrast, prominent choice theorists such as George Ainslie (24) and Gene Heyman (25) argue that the universal principles of choice that underlie ordinary behavior also explain the drug-seeking behavior of addicts. If we want to explain what people choose and do we must understand it in terms (broadly speaking) of satisfactions sought or pains avoided. George Ainslie claims that an economic theory of action must assume that the “individual is constrained to choose the option with the greatest expected reward of all those she considers.” [(2), p. 116] It is impossible for the agent to be more motivated to pursue a lesser perceived reward over a greater reward when both are available to her. The addicted person follows this pattern: her choices, like all other choices, aim at reward and are responsive to incentives. She uses drugs because they offer her more in the way of pleasure or reward than the available alternatives. Seen like this we may think that her choices are, in themselves, no more to be condemned than those of people who are preoccupied with exercising, work, stamp-collecting, or gourmet food – though like these other choices they may be criticized if they impose unacceptable costs on others or are pursued by unlawful means. Drug users, including those who are called addicts, choose to use drugs, and upon examination the explanation for what they do is of a piece with explanations of the voluntary behavior by non-addicts. This, we take it, is central to the Liberal View.

Models of addiction which propose that the user aims at the highest reward on offer face an obvious problem that choices in other domains usually do not: that of the not infrequent cases in which continued drug use incurs heavy costs, such as loss of employment, damaged relationships, legal penalties, and poor health. Additionally the initial intense pleasure that drug use delivers tends to fade over time and so it is hard to see the pleasure gained as outweighing the obvious costs. Ainslie and Heyman explain the addicted person’s chronic drug-seeking in the face of diminishing rewards and higher costs in the following way. Activities that are initially extremely highly rewarding set up inflated expectations of future reward. The promise of drug rewards in the immediate future combined with overly steep discounting of the value of other more distant rewards is further exacerbated by the toxic effects of addictive rewards on other natural rewards which drain them of the pleasure which could normally be expected from them. Drug use thus continues to promise, and they claim to deliver, more reward than the *immediately* available alternatives, even though the amount of pleasure on offer is substantially reduced and even though, were the drug user to delay gratification for long enough they would reap greater long-term rewards. This model is argued to provide a more useful and more optimistic framework than the disease model in providing directions for treatment based on positive incentives that can out-compete drug rewards.

One of us has argued elsewhere (3, 4) that the choice model and the reward account of motivation on which it rests fails to provide an adequate explanation of the actions of a small but significant subset of those who are called addicts and so fails to set aside the possibility that the disease model applies to this group. We will not rehearse all of those arguments here but we flag our concern that disease theorists and choice theorists may not be applying the term “addict” to the same group and thus that conclusions that may be warranted for the larger group of substance abusers who mature out of harmful drug use upon acquisition of new interests and responsibilities do not transfer to those hard core users with whom clinicians are concerned.

Another concern with the Choice account centers on the meaning of the term “reward” in the theory. According to this account, even where it is difficult or impossible for us or for the person concerned to identify the reward that drug-taking offers – as for example in some cases of chronic alcoholism where the physical ill effects of use are immediate and severe – ex hypothesis there must be such a reward or the person would not keep choosing to use drugs. In our view this claim is either trivial or false. We address the triviality claim here; we will provide reason to think that any substantive claim is false in a later section.

If we stipulate that *all* action aims at some reward (or relief), then the conclusion that drug users are motivated by the rewarding properties of their substance of choice follows from the fact that their behavior is intentional. Choice reveals preference. Of course, we can make this stipulation if we want. Our claims about the role of reward in addiction will then be unfalsifiable, and so of no interest, since the notion of reward is detached from its ordinary meaning and loses any explanatory value. On this technical reading of the notion of reward to say that some episode of drug use aimed at reward means *no more* than to say that it was motivated. This we do not dispute. The interesting question is whether reward in the everyday sense is what motivates drug use in addiction.

## The Liberal Account of Addiction

We see the Liberal View of addiction as arising from the picture of human motivation promulgated by the choice theorists and as gaining some warrant from it. Like the Choice theory the Liberal view says we must start from the assumption that addicts act to satisfy their strongest preferences and the driver for preferences that Foddy and Savulescu nominate is an appetite for the pleasure that drug use offers. They say “we should accept that many addicts may be choosing to use drugs because they desire drug use more than any other thing” (2010: p. 14).

Like choice theorists, Foddy and Savulescu reject the disease model and its claims that addictive action is non-autonomous. They claim that there is nothing special about the choices of those who are addicted – their ordering of values may be different to the non-addict, however, we cannot infer from this that their will is diseased or their choice-making disordered (2010: p. 14). But while choice theorists acknowledge the apparent irrationality of addicted choices and seek to explain why addicts choose what appears to be objectively worse for them over the long run, the Liberal View holds that there is no principled reason to think that addictive actions are irrational at all. Although, *some* actions performed in the course of addiction may turn out to be non-autonomous, so too may the apparently autonomous actions of non-addicts (2010: p. 15). We are not entitled to make the judgment that the bad or weak-willed choices of the addicted person are worse or different in kind to bad or weak-willed choices made by the non-addict.

Foddy and Savulescu’s target is the normative framework and assumptions that surround drug use and inform both the Disease and Moral Models. Absent the normative assumption that a life devoted to the pleasures offered by drug use is lacking in value we have no reason to suppose that the addict is lacking autonomy. Foddy and Savulescu think that if we adopt a neutral Liberal position on the values at stake we must remain agnostic on the question of the rationality and autonomy of addicts. The main contours of their argument are as follows:

- Neurobiological accounts of addiction that support the Disease model do not sufficiently distinguish the behavior of addicted persons from habitual behaviors for other non-drug-like substances, such as sugar, or activities like gambling (2010: p. 4–6)
- Addictive behaviors are not irrational, nor can we say that they are non-autonomous (2010: p. 7–8)
- It is important not to confuse any negative consequences resulting from the consumption of addictive drugs arising from cultural norms and legal sanctions against those practices, with the consequences of consumption of those same drugs absent those norms and sanctions (2010: p. 9) (A related point is a normative bias in the Disease View: the DSM, for instance, nominates as one diagnostic criterion continued use despite knowledge that it is causing “a persistent or recurrent physical or psychological problem.”).
- Once we eliminate the errors of the opposing views all we can safely say is that substance addiction involves the seeking and taking of drugs in response to strong, regular, appetitive desires (2010: p. 14).

Let us now unpack the points above in more detail. Habitual actions that aim at satisfying desires for pleasure, considered as a general category, lead to changes in neural architecture and adaptations which cement new patterns of the same behavior. The noteworthy thing about illicit drugs, say Foddy and Savulescu, is *only* that the causal pathway to neural modification is special: certain pleasure-involving receptors are targeted directly, and the intensity of the effect is typically relatively high. But many foods and non-drug-like substances also modify brain biology, they say, as well as practices such as sex or gambling.

This observation leads to an argument: if these other substances, such as sugar, cause the same kinds of brain changes, and addiction to illicit drugs is a brain disease, then regular consumption of sugar is also a brain disease; but of course it is not. Why single out illicit drugs then? Foddy and Savulescu suggest that the reason illicit drugs are thought addictive and deserving of the disease tag, is that the category emerges from “unjustifiable factual claims” based on cultural prejudices. For example, the attribution of compulsion in addiction is generated by a normative bias that is built into philosophical, political, and popular conceptions of what a life ought to contain. In particular it should not contain the selfish and destructive pleasure-seeking that addiction brings about. But, say Foddy and Savulescu, this is indeed a bias, and it has no place in deciding the criteria for addiction, *qua* a condition that allegedly compromises rational autonomy. Their view is that we do not know whether autonomy is compromised in addiction. So, they claim, we should be skeptical of claims that addicted persons are *compelled* in their behavior around the securing and taking of drugs.

Why, according to Foddy and Savulescu, should we be skeptical about the claims that the nature of addiction compromises the capacity of persons to be effective in decision-making? Again, their argument is complex, but two points they make stand out.

First, the cultural ideology around the evils of taking illicit drugs provides powerful motivating reasons to internalize a narrative that paints the addicted person as helpless and powerless to control their urges to take mind-altering substances. Indeed, addicted persons themselves utilize this conception of their situation to deflect the stigma and opprobrium attaching to this behavior. They may even be self-deceived. This would not be surprising, say Foddy and Savulescu, for this reason: “[g]iven that the average person subscribes to some version of the Lay View, the worst thing an addict could say is that she used drugs because she wanted to or because she enjoyed it.” (2010: p. 9)

Second, Foddy and Savulescu nominate a heterogeneous set of reasons, particularly from medicine and epidemiology, for doubting the claims of compulsion. Again, they say, there is a stereotypical view of drugs as causing withdrawal, but this is overstated and cannot be generalized from the key case, heroin addiction. In addition they note, with the choice theorists that most people ultimately give up their drug habit by the age of 35. And many base their drug-taking behavior or abstention around rational considerations, e.g., life choices such as pregnancy (2010: pp. 12–14). If their behavior was compelled it would not be responsive to rational considerations and ordinary life incentives.

In the light of all this, Foddy and Savulescu sum up their own view this way:

The Liberal View contains only three claims about addiction. First, we do not know whether an addict values anything more than the satisfaction of his addictive desires. Second, we do not know whether an addict behaves autonomously when they use drugs. Third, addictive desires are just strong, regular appetitive desires. (2010: p. 14)

The conclusion is that we should err on taking at face value the behavior of drug addicts – that they are rational choosers who value drugs for their rewarding properties more than they value the alternatives. Avoiding “normative bias” they say, we should accept that on face value addicts are autonomous. The Liberal account says that non-autonomy is not a *defining* condition of addiction, even though some cases of addictive behavior might turn out not to be autonomous. Addiction on the Liberal View is a matter of acting on one’s strong appetitive desires for pleasure, and that is all.

The Liberal view developed by Foddy and Savulescu can escape the charge of triviality directed at the choice account, insofar as it assumes that what motivates drug use in addiction is pleasure and it characterizes pleasure as “[a] conscious sensation produced by the brain that has the quality of being pleasant, satisfying, or enjoyable” (2010: p. 19). Central to the Liberal View, is the claim that addicted persons respond to incentives – that is what justifies the *prima facie* assumption of autonomy – and the driving incentive much of the time is pleasure (Foddy and Savulescu do agree that drug use may fail to deliver on its aim of fulfilling pleasure.). We will return to the question of responsiveness to incentives in addiction later in this paper. Our main interest here is the role of pleasure in addiction. Let us be clear: we do not question the claim that a life devoted to pleasure-seeking may be autonomously chosen. Nor do we deny that some of those who are called addicts are autonomously choosing a life centered on drug pleasures or that many of the harms suffered by drug users are the result of illiberal social and legal policies which stigmatize such pleasures (though we note this does not apply to the very significant harms caused by legal and socially approved drugs like alcohol). What we do deny is that pleasure or reward plays the central motivating role assigned to it by Choice theorists and by Liberal accounts in an important set of cases and these are precisely the cases where we have most reason to question the autonomy of the addict.

Our self-report data suggest strongly that we should construe the role of pleasure somewhat differently to how the choice accounts would have it, especially when it is conceived as part of a narrative dynamic. The role of pleasure in addiction must be understood as changing over time. While a strong desire for pleasure plays a crucial role for many, perhaps most, people in establishing addiction, it is not so clear that pleasure or the expectation of pleasure plays this role in maintaining addiction. If it does not and we cannot easily construe the behavior of the addict as aiming at reward then there will be reason to question each of the three claims made by the Liberal View.

In Section “The Diachronic Value of Pleasure in Addiction” we present our self-report data to support our claims around the more nuanced understanding of pleasure in addiction. Before that, however, we respond to the claim made by Foddy and Savulescu that the self-report data from those with addiction problems is unreliable.

## Can We Rely on Self-Report Data to Understand the Role of Pleasure in Addiction?

Foddy and Savulescu claim that because of the taboo nature of addiction, it becomes, according to those who reject self-report data, “…*impossible* to obtain *honest* accounts from addicted persons themselves…” (2010: p. 3, our italics). They say, “[t]here is enormous social pressure for addicts to provide an alternative explanation for their drug use” [(1): p. 9]. This echoes a similar observation by Dalrymple (5) who has said that when working as a psychiatrist, he was always struck by how differently users described their addiction to him (in terms of suffering and involuntariness) compared with what they would say to their peers in the hallway (in terms of pleasure). The claim is that addicted persons will be reluctant to express to professionals and others the pleasure-incentive that is really driving their addictive actions. This claim presupposes that addicted persons are being honest and truthful with their peers and not with professionals.

While it is plausible to suppose that socially available narratives of addiction influence what users say to clinicians, courts and other concerned parties about their drug use, we think that the claim that it is impossible to obtain from them honest accounts of their motivations for use is overstated, unfair to those who seek help for their drug use or who participate in research projects, and lacking a solid evidential basis.

We suggest that: (i) Addicted persons may be ambivalent about their using and this will be reflected in differing accounts given to different groups. (ii) What they say to peers is not obviously more reliable than what they say to professionals from whom they have sought help, but even if it is, the data that we have collected is not subject to these biases. (iii) There is also social pressure on many people struggling with addiction to *remain* users when they would prefer to quit, or to use more than they want to, and this social pressure plausibly influences what they say to peers. (iv) We should distinguish between the heat of the moment and the cool reflective moment in determining what it is that people really prefer.

First, we think that insofar as users express different attitudes to different groups this may reflect genuine ambivalence about their drug use, as different considerations are brought to the fore. When with family the damage done to family relationships and the hurt suffered by those near to them will be more salient than it is with peers. With professionals damage to health and to long-term prospects comes to the fore. With peers, the pleasures induced by the drug and its social aspects will be most prominent. The question, then, is not whether the individual is lying to one of these groups. The question is, of the accounts they give, which, if any, should be privileged in providing reliable testimony concerning what motivates their drug use. Given the expression of different attitudes to different groups, attitudes underpinned by a rationale responsive to that context, there is no *a priori* reason for thinking one of these groups is privileged as the group to receive the truthful account. In particular we do not know whether an addicted person’s statement to a fellow user misrepresents their understanding of what motivates them.

Second, the responses to our own study provide reasons to doubt that addicted persons are honest with their peers (a context where the taboo of taking drugs for pleasure does not operate) and not with those professionals with whom they are engaged. Our questionnaire reflected none of the normative biases Foddy and Savulescu identify, and we made clear to the participants that our role as philosophers (not treatment professionals), oriented us to an interest in their story, and their experiences. Our open style of questioning was designed to avert any sense of being judgmental and the semi-structured nature of the interviews meant that we maximized the possibility of eliciting clear and reflective accounts of respondents’ understandings of the role pleasure played in their addictive experiences. Respondents repeatedly stated that they wanted to be honest with us, that they enjoyed the conversation and felt listened to. Sometimes they asked us directly if we wanted the socially accepted explanation or if we wanted to hear what they really thought. It became clear that respondents were not reluctant to express to us the nature of their using, its extent, and the kinds of incentives that drove this behavior, including pleasure. We will explore their reflections on pleasure in the next section.

Third, some of the responses we collected suggest that we should be cautious about privileging what they say to their peers over professionals and others, in seeking to explain their behavior. Social pressure cuts both ways and many long-term addicts live in a social milieu in which using is expected and abstinence is seen as a threat, an implied criticism, or socially unacceptable.

The other addicts aren’t really …they don’t want to see someone get on with their life “cause then… oh this is what I think, then… it’s saying to them, may be you can do this but they don’t want to… they’re comfortable. I don’t know, it’s kind of like misery loves company… you can have so many friends when you’re miserable and everybody wants to hear all your problems and they’re all so consoling you know but sometimes I wonder if they’re not being patronizing and they really like to… “cause I notice when I’m going well, no-one’s that happy and it’s like no-one wants to give you a shot when you’re hanging out but when you’ve been clean for six months everyone wants to give you a shot, it’s things like that I’ve noticed, you know. (R67)”

That’s another big step because all my so-called friends are down here and to leave them is going to be hard, but they’re not really friends anyway, they’re just acquaintances through pubs and drugs, that’s pretty much it. So, yeah, to leave them it’s going to hurt them, but it’s probably not going to hurt me as much as it’s going to hurt them, but what can you do, you’ve got to get rid of the old people, you know what I mean? (R5)

He’s [his boss, who also has a drinking problem] always ringing up to come to work, even if I have a day off – “Are you going to come tomorrow? I’ll even come and get you.” “Yeah I’ll be there, I’ll be there.” And he’d say “You got any beers in your bags?” and I say “No.” I know I got beers in the bags; as soon as I get to work I’ll open a beer, [and] by lunchtime he’s looking at me going “fuck it,” let’s go and get a beer, and I’ve already had six by then and I’m thinking oh, I don’t really need another one but I go with him and then I might have another six that afternoon. (R6)

Finally, and in response to the preceding considerations, it is common in philosophy and in common-sense, to distinguish between what people want and what they “really want” or value and the differential responses may in part reflect this distinction. Gary Watson describes a person’s values as: “…that set of considerations which he – in a cool and non-deceptive moment – articulates as definitive of the good, fulfilling, and defensible life” [(6), p. 105]. However, as Watson points out, our valuational system and our motivational system may come apart. Another related distinction is between people’s experiential interests and their critical interests [(7): p. 201]. The former are satisfied when a person’s present inclination for certain kinds of felt experiences is met. Desiring a warm bath and lying in it, having a wish to smell roses and smelling them, having a yen to hear Bach and listening to it, all count as examples. Critical interests, by contrast, are not tied either to the present, or to any kind of feeling. That one’s treasured antique violin is passed down to a grandchild, or that one’s standing in the community as a decent citizen is recognized would count as examples of critical interests.

These distinctions provide an alternative explanation to self-deception or pressure to adopt socially acceptable narratives of addiction where there are instances of different content or emphasis in what is said to professionals and what is said to peers. When addicted persons are with their drug using peers, attention-grabbing drug cues abound. Their experiential interests or immediate urges dominate their attention and what they say about drug use then is much more likely to be, as it were, in the heat of the moment. In a more reflective moment when their critical interests come to the fore, such as when they are with a therapist or a researcher, they are likely to express a measured assessment of their drug use which encourages them to describe how it impacts on their life extended over a longer period than the time it takes to make the next score.

Foddy and Savulescu’s reason for dismissing the possibility of reliable first hand reports is the taboo nature of drugs and pleasure that supposedly prevents the addict from delivering an honest appraisal of their drug-related activities to those outside their peer group. As we’ve shown there is reason to think that this assessment is unduly pessimistic – especially surely as applied to alcohol. If that is right then any claim that we should privilege what users say to their peers over what they say to professionals and others must turn on privileging experiential interests and synchronic desires for pleasure over critical interests and diachronic values, and this is a matter on which the Liberal view must remain agnostic. While both perspectives must be taken seriously, and are equally important in understanding addiction, in our view the persistence of grief, regret, and internal conflict in many of our subjects (including many alcoholics) provides at least a *prima facie* reason for privileging their critical interests.

## The Diachronic Value of Pleasure in Addiction

While the self-reports of those who are addicted cannot tell the whole story of the role of pleasure in addiction, self-report data provides a valuable insight into the changing role of pleasure over the course of addiction. In this section we will draw on material from qualitative interviews with 69 opioid and alcohol-dependent subjects in order to counter what we think is the overly narrow understanding of pleasure in addiction assumed in the choice accounts. As we have said, we agree that a life of pleasure could be autonomously preferred and that some user’s lives may be autonomously structured around the pursuit of drug-related pleasures. Most of our subjects did not characterize or experience their lives or their drug-seeking as autonomous in this way. However, our interviews revealed a nuanced and changing role for pleasure across the course of addiction.

On the basis of our interviews we can distinguish three subgroups of users: the first group said that pleasure was their main motivation for using substances. On the Schwartz Value Questionnaire they scored “hedonism” as their most important value. But this simple nomination disguised an important aspect of their self-understanding in relation to the incentive pleasure gave them. Pleasure was, for them, intensely motivating, but they realized that in the long run the damage their drug use caused had the effect of hindering their goal of a hedonistic life. They were disposed to stop their consumption for hedonistic reasons, and yet found doing so beyond them. The Liberal account argues that even in these cases where pleasure is the only value at stake, there is no reason to think one’s short term appetites must align with one’s long-term hedonistic project to maximize the rewards of consumption over time. What they say is in line with the Choice model: at the moment of consumption, it is the appetitive reward the person most wants, and that although this may count as weak-willed it is not *prima facie* non-autonomous. Our view is that, on the contrary, the extraordinary difficulty some subjects face in orienting themselves toward the pleasure they both want and value most signifies an important loss of control – of self-authorship. We make good on this claim in a later section.

The second group consisted of people who cited pleasure as the initial reason for consuming; over time, however, after repeated use, the pleasurable effects of the substances they were taking faded out and pleasure was no longer their main motivation for use. The third group claimed to have never really experienced pleasure from using. For both the second and third group their ongoing motivation to use drugs was something of a mystery to them. They explained it by reference to addiction – which they seemed to experience as a motivating force distinct from any interest in or expectation of pleasure.

### Pleasure all the way through, but a pleasurable life is more than using

We identified a sub-group of users who acknowledged they were motivated primarily by pleasure. Still, even for this group our data shows that the Choice model may be too simplistic. Those who valued hedonism did so based on an understanding of that notion that was broader than just the “instant pleasure” derivable from substance use; their sense of the value of pleasure was *diachronic* in nature.

A hedonistic lifestyle, as understood by many of those we interviewed, is not reducible to a narrowly focused pleasure-seeking or seen in terms of the aggregation of a set of pleasurable experiences. Only a minority of the respondents described themselves as pleasure seekers in this narrow sense, and even they were skeptical about the contribution of substance use to their hedonistic lifestyle in the long run. One person, who described himself as hedonistic, made clear that substance use was only part of a hedonistic life. Other users described how drug use can conflict with other primarily hedonistic values, such as holidays and material goods, which nevertheless require planning and a diachronic perspective at odds with the synchronic focus induced by substance use.

I just enjoyed life and work but life more than work (…). I think I wanted to be successful. I was very hedonistic. You know I wanted the right clothes; I wanted to eat in the right restaurants and be with the right people, go to the right parties and that sort of thing. (MHE 001)

When you’re drinking you’re just thinking of the moment, you’re not thinking of anything else sort of thing, anyone or anything in particular you know, you’re just thinking about having a good time and a laugh and a joke maybe with a couple of friends that you’re with or something like that, but you’re not… it’s not as if you’re sitting there talking about planning and buying a house or what are we going to do… plan a holiday to go overseas next year or something like that. (R32)

One young female alcoholic stated that although she was doing many nice things in her life (including a job she enjoyed, and frequently attending festivals), due to her excessive alcohol use, she was not able to remember many of those enjoyable things and that her alcohol use was also frequently spoiling enjoyable occasions. Another user described herself as a “willing addict”; she claimed that all she had ever wanted to become in life was an addict. However this seemed tightly connected with a kind of status she had within her using and dealing family gained by her ability to be able to get every prescribed medication she wanted from the time she was a minor, rather than from the pleasures of use itself.

Additionally most of our respondents were highly skeptical about the possibility of long-term use without significant negative consequences.

Well don’t get me wrong, I love using mate. If I could use successfully I would. I’d still be using. I love using; I just don’t like the shit that comes with it. (R50)

Another respondent described it as follows:

Heroin is an astonishing thing. I will never… regret taking heroin. In fact those two years I took heroin are actually one of the best two years of my life. (P1)

Yet this respondent did decide to stop because of the negative consequences of his use. He describes the experience of coming off heroin as an extra bill he had to pay for his use, an extra hard time. Adherents to the Choice model will say that such cases make their point. It shows that people *will* stop using when the costs become too high – and of course many of those costs are a result of the unjustified normative bias which stigmatizes drug use.

In response we agree that many people do stop using when the costs rise and this may be particularly true of this hedonistic sub-group.

But others don’t even when, from their own point of view the costs are manifestly enormous – including impending death – and the hedonistic benefits are invisible. If the Choice and Liberal theorist’s claim that addicts will stop when the costs become too high amounts to the truism that addicts will stop using when their momentary drives to use no longer outweigh competing motivations they have done no more than reiterate their own account of motivation. The important issue for us is *why* some individual’s motivations are unresponsive to massively increasing costs and decreasing rewards and whether this calls into question the Liberal *prima facie* assumption of rationality or autonomy in these cases.

The assumption of the Liberal and Lay views that addicts freely choose to take addictive substances for their rewarding properties certainly has application *to a sub-group of addicted persons*. However we see that even in this group people are quite skeptical about the contribution of substances to a pleasurable lifestyle in the long run. Although they don’t necessarily regret their use and still like the effects of the substance, they acknowledge the ways in which repeated consumption for instant pleasure ultimately undermines other diachronic values and reasons that they endorse. The Liberal view accepts this latter nuance, but insists that individuals among this group remain motivated by their appetite for the rewards of their preferred substance and that we have no special reason to suppose they lack autonomy. To do so is unjustifiably to privilege their reflective preferences over their first order preferences. We think that there is an important difference between the initial motivational profile of the hedonist drug user who smoothly translates her pleasure-oriented values into action and the same person later on, utterly disabused of the belief that drug use will promote her hedonistic ends, but who is nevertheless episodically motivated to consume drugs when the cues pressuring her to do so become overwhelming – a difference relevant to the assessment of autonomy.

### Initial pleasure

This group of respondents said they used substances for their pleasurable effects, but only, or especially, at the start of their addiction. They described their initial use as a honeymoon period, until their lives began to fall apart, a period in which substance use ceased to be pleasurable:

that’s the love-hate thing I have with… when I first started, I liked the feeling but then once I got addicted I didn’t like it. And I always wanted to quit because of that. (FHE 041)

This group, for whom drug use no longer produces pleasure and who want to quit, divides between those who end up using to ameliorate the negative effects of craving and withdrawal and those for whom pleasure or relief ceases to play a useful explanatory role.

I mean some people will say oh, the drugs stopped working for me. I don’t agree, you know, I don’t believe that… I mean I think if they weren’t working you wouldn’t do them. They do. They make a person feel… (…) and then after a while, when I said it takes on a life of its own, what you get is the sort of relief that you get when you stop (…) running, you know (…) you’re really punching that last couple of Ks out or whatever (…) ‘cause you know that when (…) you get to that certain point and you get that needle into your arm and you get it you’ll be able to breathe, you’ll be able to go, oh, phew, it’s … that’s all better, it’s [like] bashing your head against a brick wall, it feels so good when you stop. (MHE 9)

While it sounds odd to portray those who use substances to relieve unpleasant sensations as living a hedonic existence, drug use for such individuals might still be the most rewarding option. The relief described by this user is indeed fully consistent with the choice model and can be accommodated by Foddy and Savulescu. Yet even where our users’ stories are consistent with the claims of the choice account we think these accounts miss something important in the phenomenology of addiction. The idea of drug use “taking on a life of its own” recurs throughout our interviews. It is the point at which drug use ceases to serve its original hedonic function and becomes detached from the user’s perceived interests, values, and desires.

We think that for a significant sub-group of users pleasure ceases to have the explanatory value attached to it by Foddy and Savalescu, and that at some point neurobiological models, such as that proposed by Robinson and Berridge (8); Berridge et al. (9), and Koob and Volkow (10), are a better fit with their reported experiences. The fit between the neurobiology, the phenomenology, and the behavior may be thought to constitute converging lines of evidence for the view we present. We do not suggest that the neurobiological evidence could be sufficient on its own.

These neurobiological models do however purport to provide an explanation of the *shifting* role of pleasure in different stages of addiction that our subjects and others describe. Although initial substance use can release a large amount of dopamine in the brain, causing intense feelings of pleasure, repeated substance use has quite a different effect. Because the brain is over-fueled with dopamine, neural changes in the reward pathways occur to restore the balance, such as the decrease of post-synaptic dopamine receptors, to overcome the effect of the substance. This results in tolerance for the substance (with less pleasure experienced), but also a higher threshold for experiencing those rewards obtained from normal rewarding activities, like food, sex, and social cooperation. Koob and Volkow (10) call this the “motivational withdrawal syndrome,” roughly, the emergence of a negative emotional state – anhedonia – that occurs after abstinence [(10), p. 217]. This state can persist for months or even years after abstinence.

But that is not the only change caused by the huge surges in dopamine release by substance use. Dopamine’s function is twofold: it primes us on the circumstances or cues in which the pleasurable event occurs, and it reinforces behavior that is directed to those goals. These effects occur because the intensity of a drug experience provides a learning signal that this reward was better than expected. On the next occasion when the same cues appear we will be more sensitive in our recognition of the type of activity generating what we have learnt, and we will be disposed to pay attention and direct our behavior accordingly (11, 12). The huge amount of dopamine release works as a Trojan horse that overtakes the reward-related learning process and creates long-term associative memory processes directing a person to further substance use [(13). p. 575]. Becoming hypersensitive for drug-associated cues then occurs mostly in the absence of subjective feelings of pleasure. Thus the increasingly addicted person continues to want a substance they no longer have a strong liking for. Repeated substance use increases compulsive wanting, or craving, and at the same time diminishes experienced pleasure. Normally we want what we like, and we like what we want, but Berridge (14) has shown that these systems operate through different neural pathways. It is not so much the pleasurable effect (the liking) that drives the addicted person, but the reinforcing, conditioned learning aspects of dopamine driving the behavior (15).

Summing up, there is a major strand of research that argues that the neurobiological effects of sustained drug use help to understand and characterize the function of pleasure for this group. This group learns to continue wanting a drug that has ceased to generate for them the pleasure it originally had. This is not just a “very strong appetite” for pleasure as this is normally understood. It has compulsive elements divorced from any person-level expectation of pleasure, in that it captures and monopolizes the addicted person’s attention making it extremely difficult for them to focus on and pursue other more valued activities.

We acknowledge that the science is far from settled, so it pays to be cautious in recruiting data to support philosophical theorizing. Philosophers lack the expertise to adjudicate between positions within the neuroscience of addiction. But we do not in any case claim what Foddy and Savulescu are especially keen to deny – that this research establishes that addiction is a brain disease or that addictive action is somehow not intentional. We take no position here on whether addiction is a brain disease (We think that some of the common arguments against the disease claim are bad arguments, but that’s another story.). We do not think that an argument that the autonomy of addicts is impaired depends on establishing a disease model or upon showing that addicts do not intend and choose those of their actions that are motivated by their drug-related urges.

While it is clearly true, as the choice model emphasizes, that the particular actions an addicted person undertakes in procuring and consuming drugs are responsive to a variety of contingencies, we think this flexibility is not as significant as proponents of such models have it. In particular it does not show that drug consumption is not in some sense compelled, or that it must be the most rewarding synchronic option available, at least on any ordinary understanding of the notion of reward [see (3) on this point]. On a view which sees wanting and liking as dissociable and dissociated in many long-term users, drug use ceases to be chosen in the sense proposed by the choice models, that is, as *rationally responsive*, either globally or locally, to an evaluation of the rewards on offer. The *means* taken to drug use may indeed be flexible and responsive to local contingencies and so drug use can be delayed or moderated in some circumstances, but the *goal* itself seems to be a stubborn feature of their psychology. In the hard core user it is relatively impervious to reflection, choice, and control, even when it is clearly highly dysfunctional. We think that this is an important feature of addiction. Drug use becomes, as one of our respondents put it, like a chore or an “obligation.” It becomes something they have to do but that they no longer enjoy or understand themselves doing. Here is a representative sample capturing this idea:

Yeah but now it’s just… it’s not even fun anymore really, it just sort of becomes a… I don’t know, more or less like a chore I suppose but yeah I just… I want to get away from it. (R29)

It’s… there was reason, early part, until I came to understand why I was behaving the way I was behaving. So in… no, not now. No. There’s no reason. (R39)

[W]hen I was 20, 30, when I was 40 my drinking was good, I had good times on the drink, from when I was 50 to 60 just… I’m just drinking for nothing (…) I’m just drinking for drinking sake now. (R24)

Now Foddy and Savulescu may counter that we see the same phenomenon in the other kinds of cases they give. Perhaps repeated high consumption of sugar or repeated gambling has the same effects in some people and so they feel driven to consume or to gamble even though they say they no longer enjoy it, and even though it has disastrous consequences which they certainly do not enjoy. If this were to be the case we would not see it as reason either to reject those neurobiological or phenomenological accounts which accept the liking/wanting dissociation in addiction, or to become skeptical about the category of addiction. The relevant behavior is addictive even if it responds to substances or stimuli which do not usually pose a risk of addiction^2^.

### Never experienced pleasure

Summing up, the groups we have described so far report that although the drug use fulfilled a certain role for a period of time, at some point this ceased to be the case. It ceased either because tolerance led to a loss of pleasure, or because, for more complicated reasons, their drug use could no longer be rationally or successfully incorporated as part of a more sophisticated hedonistically motivated lifestyle. They developed a love-hate relationship, or simply a hate relationship, with their drug of choice as the pleasure diminished and the costs became too high.

We can now distinguish a third group who don’t describe any feelings of pleasure or hedonism when using drugs. Some within this group emphasized the strong physical dependency that came with their consumption:

[A] lot of people talk about a honeymoon period on drugs. I can’t remember a time like that, I can remember starting drugs and pretty much straight away trying to stop all the time. Like I know people talk about that it was nice and exciting and it was a carnival at the beginning but I didn’t find it like that (…). I hardly even remember starting drugs, I mostly remember trying to stop all the time. (R47)

Others within this group described the use of substances as a way to feel normal, as a painkiller, or self-medication for psychological problems. One young woman described how she always felt she didn’t have a right to belong in the world the same way other people do, she would stare in the mirror for hours, trying to figure out who she was.

[U]sing heroin made me feel normal, it took that away, so I didn’t feel bad about it at all, I thought I’ll do anything I can to get it, I don’t mind if I have to work [in prostitution] and I thought that it was the only thing that would help but of course it’s taken everything away from me now (…). Yeah I didn’t use it to have fun I used it to feel normal, then it turned into just an addiction. (R22)

Addiction is seen by many of our subjects as a motivating force that is separate to and distinct from the desires for pleasure or relief or acceptance that originally motivated their drug use and as undermining both their pleasures and their plans. In the light of the costs their drug use imposes on them we think that, for at least a subset of users, pleasure, and reward do not explain continued use.

## Autonomy and Addiction

In light of the distinctions of the role of pleasure in addiction we think it appropriate to respond more fully to an argument from Foddy and Savulescu regarding addiction and autonomy. We would claim that the evidence of dissatisfaction and repeated failed attempts to quit calls into question the autonomy of the addict. We do not assert that it calls into question a *substantive* conception of autonomy, for we are not here making any normative claims about the irrationality of seeking pleasure through substances over more healthy activities. We agree with Foddy and Savulescu [(1): p. 8] that in testing whether addiction threatens autonomy the correct conception is a procedural account. Then the question of whether autonomy is threatened has nothing to do with the *content* of the desire one acts upon, yet fails to identify with or endorse. Whether autonomy is threatened is a matter of whether the machinery of the will – involving the interplay between the motivational and valuational systems – functions properly. Foddy and Savulescu claim that addiction does not diverge in any significant way from many other phenomena in which agents repeatedly regret past actions. They write (p8)

Human beings make choices they regret, sometimes even repeatedly. There may be an ideal conception of autonomy, according to which making choices in the knowledge that one will regret them later, is non-autonomous. But telling us that addiction is non-autonomous in this sense is telling us very little: It is not distinguishing it from ordinary cases of weakness of will.

But, on the contrary, we think severe cases of addiction are not like the ordinary cases of weakness of will they have in mind. To explain this more fully we invoke a tripartite distinction between wanting, liking, and valuing. In ordinary cases of weakness of will wanting combines with liking in opposing the agent’s best judgment. When I eat chocolate though I’m on a diet or snuggle up in bed instead of going for an early morning swim in accordance with my fitness regime I am doing what I both want to do and enjoy doing at that time though I think that all things considered I should be doing something else and even though I know I will regret it later. But I *like* chocolate and warm beds. If I did not, my actions would be puzzling indeed.

That we can describe these cases as weakly giving in to one’s desires for chocolate or for comfort makes sense partly because the pleasure or reward competes against the value judgment^3^. Are cases of addiction just like this? If they are, then we do indeed have reason to be skeptical about the category of addiction, since in the story just given we have no reason to conclude that I am addicted to chocolates, warm beds, or whatever else.

However, if we take the self-report data from addicts seriously, as we have argued that we should, this is not true of at least a subset of addicts for whom even the immediate outcome of their consumption is dominated by pain and regret. For this group, pleasure (or reward) and the expectation of pleasure (or reward) – that is, what they *like* – has dropped out of the picture^4^. An appetite toward pleasure or reward does not explain their actions. Here the competition is between valuing and mere wanting. You may want something you neither like nor value; furthermore you may want it so strongly that you simply cannot stop thinking about or successfully inhibit the automatic action tendencies that arise in response to environmental cues, and any attempt you make at synchronic self-control will eventually fail. Both strength and persistence of wanting and the opposition of wanting to liking or valuing, are important elements of what distinguishes weakness of will – even persistent weakness of will – from compulsion. Autonomy comes in degrees and while there will be borderline cases we claim that at least some hard cases of addiction are clear cases of compulsion rather than weakness of will – even chronic weakness of will – or unthinking habit^5^. If the distinctions outlined here are correct they suggest that addiction cannot be as readily assimilated to everyday moral experience as proponents of the Liberal View suppose and places the onus back on them to explain, consistently with their view, what has gone wrong in such cases.

We see a significant problem with the Liberal position of neutrality between synchronic and diachronic perspectives in an account of autonomy. The Liberal position does not want to privilege the satisfaction of our reflective desires over the satisfaction of desires formed in the heat of the moment. We of course agree that the satisfaction of momentary desires for such things as food, sex, or drugs can often contribute value to someone’s life, and that we should not automatically assume that a person who prioritizes synchronic pleasures lacks autonomy. A view which prioritizes reflective preferences can accommodate the endorsement of the satisfaction of synchronic desires as autonomous and can also describe when they become non-autonomous. But what, on the neutral Liberal view, could count as impaired autonomy at all? Foddy and Savulescu agree that it is possible some addicts lack autonomy. Which addicts, and what would they have to lack *qua addicts* for Foddy and Savulescu to count them as having impaired autonomy?

Let us briefly sketch a reason for thinking that the procedural account we favor is to be preferred over neutrality. Who or what can be autonomous? It seems to us that a condition of autonomy is diachronic agency. Purely synchronic agents, e.g., very young children or deeply amnesic patients, cannot be autonomous. You need to be capable of remembering the past and projecting yourself into the future – you need the capacity for mental time travel – in order to be autonomous. But of course mere access to your past and the capacity to predict the likely future is not enough for autonomous agency. As two of us have argued at length elsewhere both planning and diachronic self-control are fundamental to the construction of the kind of unified agent who can properly be held responsible for their actions (16–19). The importance of diachronic capacities and perspectives in the construction of an agent who is even so much as capable of autonomy or failures of autonomy does at least suggest a reason for privileging the reflective perspective in identifying when autonomy is impaired and to what degree. In severe cases of addiction the radical impairments to diachronic agency can be understand only when we see the individual as failing to unify their agency in accord with their reflective selves.

## Conclusion

Pleasure seems to play a significant role in addiction though this diminishes across time and users become increasingly resentful of, or despairing of, the effects of their substance use on their capacity to realize other values. The Lay and Liberal Views are of course right that a desire for pleasure can play an important role in explaining consumption in first-stage substance use, though this excludes the category of users who self-medicate. But in the long run the influence of substance use on health and on social relationships seems, to many users, not to be worth the trouble, especially when the early pleasurable effects fade. Yet, for a significant number of users in this group, *the loss of pleasure does not result in a cessation of addictive use*. Even for the people who seemed to want to pursue a life of hedonism, substance use has only a temporary role in this. Although our respondents did not deny the pleasurable effects of substances during the early stages of their use, they were mostly quite skeptical about the pleasurable effects in the long run. This seems to us to present a problem for accounts that depend on an assumption that the desires for pleasurable rewards continue, more or less in the same form, throughout different phases of addiction. The Lay View seems particularly taken with this assumption. It also presents a problem for the idea that we should err on the side of assuming that those who are called addicts are rational choosers who value drugs for the pleasure they produce more than they value anything else.

Our interviews combined with other data call into question each of Foddy and Savulescu’s three assumptions: First, while we do not know for certain whether any addict values anything more than the satisfaction of his addictive desires, the clear unhappiness of many of them with their drug-taking lifestyle and their repeated attempts to quit suggest that they do.

Second, the same evidence of unhappiness and failed attempts to quit also calls into question the autonomy of the addict. Many are no longer motivated by what they like about drugs and so cannot be characterized correctly as acting weakly in the way we might characterize someone who regrets eating too much chocolate (for pleasure) on some occasion. The addicted person is not weak of will in that sense. The stubborn resistance of their goals to their reflective judgments is not properly explained by assimilation to ordinary cases of temptation where for the most part we do manage to act in accordance with our judgments.

Third, addictive desires appear to shift from being, “just strong, regular appetitive desires” to desires which have lost the ordinary connection with reward. With or without any normative bias that may play a role in shaping an addicted person’s preference structure, it is misleading to portray people struggling with addiction simply as motivated by strong appetites for pleasure. For the last group we identified it looks false. Those people never experienced the claimed rewards. Others struggle to quit despite extraordinarily heavy and increasing costs. Many of our respondents continued using in the face of costs which were not comfortably relegated to a distant and discounted future but were rather experienced by them daily, including at the point of use – such as pain, serious, and disabling health problems, and very credible risk of death. In our view it does not appear that such users are acting autonomously on the basis of a strong appetite for pleasure, or that their motivation conforms to the supposed universal principles underlying the choice model. If the choice view or the minimal Liberal view licenses agnosticism on the issue of whether such individuals suffer impaired autonomy or not it leaves us wondering what, if anything, a clear case of impaired autonomy could be.

As one of us has argued elsewhere, to insist either that such users *are* motivated by pleasure or reward or to make this the default assumption makes the Choice model stipulative rather than genuinely explanatory of a range of cases (3, 4). This is a pity since both the choice model and the Liberal view to which it gives rise have insightful things to say about addiction and the role of pleasure in establishing and maintaining it.

## Conflict of Interest Statement

The authors declare that the research was conducted in the absence of any commercial or financial relationships that could be construed as a potential conflict of interest.

## Footnotes

^1^All 2010 in-text references to Foddy and Savulescu will be to this work.

^2^We think that behavioral evidence and phenomenological report strongly suggest both the dissociation between wanting and liking in human addicts that Berridge claims to have found in rats, and the monopolization of attention and cue-driven action in addiction. Certainly the claims about monopolization of attention, cue sensitization, and cue-driven behavior in addiction are uncontroversial and have been established in many psychological studies that do not rely on neuroscience. Addiction neuroscience aims to uncover the neural mechanisms that drive behavior. Perhaps the work we have cited – though it is certainly in the mainstream – does not successfully uncover those mechanisms and explain the observed phenomena. It might explain too little: there could be other relevant pathways that explain addictions not well explained by the dopamine theory. It might on the other hand explain too much. This seems to be the thrust of much of Foddy and Savulescu’s discussion of the data. They say the same responses have been observed for a wide range of non-drug substances so either we must say that people may be addicted to all these other substances (a reductio) or the research simply describes normal responses to pleasure and does not serve to support the view that addicts are in any way impaired (qua addicted). Perhaps they are right; more work needs to be done to test this view. But there is at least a respectable body of opinion that people may form addictions to non-drug substances such as sugar and to activities such as gambling, with similar behavioral features and consequences (mutatis mutandis) to drug addictions. Whether this will extend to include such apparently innocent substances such as water and milk as Foddy and Savulescu suggest remains to be seen since the studies they refer to have not, to our knowledge, been replicated.

^3^This is not the full story of weakness of will. See Kennett (20) for a detailed account.

^4^That is, the expectation of pleasure at a personal level. Perhaps the system “expects” pleasure – in the sense laid out by the neurobiological accounts. If so the explanation of action is sub-personal and as argued elsewhere (3) this would be inconsistent with an important feature of choice accounts – that the addict’s action is susceptible to a rationalizing explanation, in terms of their beliefs and expected reward.

^5^Note: this is *not* to suggest that addicts lack autonomy altogether or that it is impossible for the compelled addict to reduce or quit – since they may be able to avail themselves of techniques of diachronic self-control. Nor is to suggest that *chronically* weak-willed individuals are not also lacking autonomy. We think they are to the extent that they become synchronic, disunified agents – see discussion below and see also Levy (21) for an interpretation along these lines of the failure of autonomy in addiction. There is no space here to provide a complete description and defense of the distinction we have outlined between weakness of will and compulsion: for a book length treatment see Kennett (20), and for particular application to the case of addiction see Kennett (3, 22) and Kennett et al. (23).
